# Adverse trajectories of mental health problems predict subsequent burnout and work-family conflict – a longitudinal study of employed women with children followed over 18 years

**DOI:** 10.1186/s12888-016-1110-4

**Published:** 2016-11-08

**Authors:** Wendy Nilsen, Anni Skipstein, Evangelia Demerouti

**Affiliations:** 1 Department of Mental Disorders, Mental and Physical Health, Norwegian Institute of Public Health, Postbox 4404, Nydalen, 0403 Oslo Norway; 2 Work Research Institute, Oslo and Akershus University College of Applied Sciences, Postbox 4, St. Olavs plass, 0130 Oslo, Norway; 3 Technische Universiteit Eindhoven, Eindhoven, Postbox 513, 5600 MB Eindhoven, The Netherlands

**Keywords:** Mental health, Trajectories, Burnout, Work-family conflict, Women, Parents, Mothers, Longitudinal

## Abstract

**Background:**

The long-term consequence of experiencing mental health problems may lead to several adverse outcomes. The current study aims to validate previous identified trajectories of mental health problems from 1993 to 2006 in women by examining their implications on subsequent work and family-related outcomes in 2011.

**Methods:**

Employed women (*n* = 439) with children were drawn from the Tracking Opportunities and Problems-Study (TOPP), a community-based longitudinal study following Norwegian families across 18 years. Previous identified latent profiles of mental health trajectories (i.e., High; Moderate; Low-rising and Low levels of mental health problems over time) measured at six time points between 1993 and 2006 were examined as predictors of burnout (e.g., exhaustion and disengagement from work) and work-family conflict in 2011 in univariate and multivariate analyses of variance adjusted for potential confounders (age, job demands, and negative emotionality).

**Results:**

We found that having consistently High and Moderate symptoms as well as Low-Rising symptoms from 1993 to 2006 predicted higher levels of exhaustion, disengagement from work and work-family conflict in 2011. Findings remained unchanged when adjusting for several potential confounders, but when adjusting for current mental health problems only levels of exhaustion were predicted by the mental health trajectories.

**Conclusions:**

The study expands upon previous studies on the field by using a longer time span and by focusing on employed women with children who experience different patterns of mental health trajectories. The long-term effect of these trajectories highlight and validate the importance of early identification and prevention in women experiencing adverse patterns of mental health problems with regards to subsequent work and family-related outcomes.

## Background

Mental health problems, such as depression and anxiety, are of major public health concern [1–3]. The burden of mental health problems may lead to several adverse consequences for the individual and their family, as well as the society as a whole. The personal costs are incremental – including reduced life quality and functioning [1, 3, 4]. Due to the increasing numbers of employed parents in the labor force the last decade, and the extra stressors associated with parenthood [5], there should be a larger focus on mental health in parents and potential adverse work- or family-related outcomes.

While parenthood is related to well-being and meaningfulness, findings also suggest that parenthood might increase the risk of mental health problems through mechanisms such as sleep deprivation, fatigue, and financial strains [5]. Still, few studies have examined the development of mental health problems in parents throughout the entire child-rearing phase [6]. Importantly, the child rearing phase spanning from early childhood to adolescence might involve different transitional stages characterized by different events and challenges, which might affect the parent in addition to the child. Using a person-centred approach, where the goal is to categorize individuals based on their common and divergent phenotypic patterns across time, would be beneficial for identifying adverse patterns of symptom levels and their development (i.e., trajectories) [7]. This approach is particularly recommended when different temporal patterns are assumed to have dissimilar implications for individual outcomes and when trajectories are assumed to systematically differ across individuals [8, 9], which often is the case with mental health problems. We focus specifically on the outcomes burnout and work-family conflict, as these have not been studied in the past in relation to maternal mental health trajectories although they represent detrimental effects on individuals, their family and work [10–19].

### Burnout

Due to recent changes in global economy, such as heightened job insecurity and increased demands due to financial crises, burnout is likely to represent a major public health problem over the years to come. Burnout has been defined as a psychological syndrome of exhaustion, feelings of cynicism and detachment from the job, and a sense of ineffectiveness and reduced personal accomplishment, in response to chronic stressors on the job [20]. The current study uses the Oldenburg Burnout Inventory (OLBI), which measures feelings of *exhaustion* and *disengagement from work*, applicable to most occupations and includes both *negatively* and *positively* worded items for each dimension [21, 22]. *Exhaustion* is defined as a consequence of prolonged and intense physical, affective and cognitive strain, as the result of continued exposure to specific working conditions (or stressors). The OLBI covers not only affective (e.g., emotionally drained), but also physical and cognitive aspects of exhaustion (e.g., need of long resting time). This coverage makes OLBI more applicable both to those workers who perform physical work and to those whose job is mainly about processing information instead of dealing with people. *Disengagement from work* refers to emotions regarding the work tasks (e.g., uninteresting, and “disgusting”), as well as to a devaluation and mechanical execution of one’s work, and represents an intensive reaction in terms of an emotional, cognitive, and behavioral rejection of the job and it delineates an occupational disillusionment [23].

Burnout has been associated with adverse health outcomes such as poor health behavior, somatic and mental health [11, 20] as well as organizational related outcomes such as long term sick leave [10], poor job performance [12, 20] and low cognitive functioning [13] and is thus important to prevent. For instance, Peterson, Bergstrom, Demerouti, Gustavsson, Åsberg and Nygren [24] reported that higher scores on the exhaustion component of the OLBI were associated with future long-term sickness absences (≥90 days) in female professionals. Long-term sickness absences were collected 44 months after the measurement of burnout, which underscores the important detrimental effects of burnout.

### Work-family conflict

Work-family conflict is typically defined as “interrole conflict in which the role pressures from the work and family domains are mutually incompatible” [25–27]. The perceived levels of work-family conflict have potentially increased the last decade due to increasing numbers of employed parents in the labor force. Meta-analyses report significant associations between work-family conflict and adverse work and health-related outcomes [14–19]. In addition to adverse individual outcomes, findings also indicate that work-family conflict impairs the relatives of the employee [28, 29]. For instance, recent findings suggest that work-family conflict significantly compromise optimal parenting and partner relationship satisfaction for employed mothers [28]. By identifying which mothers are at the highest risk for work-family conflict, one might be able to develop suitable intervention efforts to be able to prevent or reduce the conflict between work and family.

### Trajectories of mental health problems and subsequent work-family conflict and burnout

Mental health problems are related to both burnout [30] and work-family conflict [15, 16, 31]. Still, very few studies have examined these associations in a longitudinal person-oriented framework focusing on mental health problems. There are, however, a couple of exceptions. For instance, a 10-year longitudinal study of university students reported high depressive symptom trajectories to be associated with subsequent burnout compared to moderate and low symptom trajectories [32]. No studies, as far as we know, have examined these relationships (between mental health and burnout or between mental health and work-family conflict) over the longer child-rearing periods in employed parents. One exception is a recent study which reported prospective reciprocal associations between psychological distress and work-family conflict in employed mothers of children followed from over 5 time points over a period of 8 years [31]. Albeit an important study, we still do not know how different *trajectories* of mental health problems across the entire child developmental period predict subsequent adverse outcomes.

Most studies examining parent’s mental health are from industrialised countries with modest welfare benefits (e.g., United States, United Kingdom and Australia) [33]. Parenthood there might be very different from the Scandinavian setting, which have a more generous welfare system promoting flexible work-family solutions, long maternity leave and generous social benefits [33, 34]. Except for the Scandinavian countries, which have extensive national work-life policies, work-family balance is perceived as within one’s private responsibility in the majority of the world. A study of several European countries reported that the Scandinavian countries score higher on state, workplace and family support compared to other countries, and these kinds of support had a direct and buffering effect on satisfaction with work-life balance [34]. Thus, it is interesting to examine how parental mental health are related to work-family conflict and burnout in the Scandinavian setting having higher degree of social security, which might buffer such adverse consequences.

In the current study, we aim to examine how trajectories of mental health problems (i.e., symptoms of anxiety and depression) in women with children followed over six time points (from 1993 to 2006), differ in levels of burnout (i.e., exhaustion and disengagement from work) and work-family conflict 5 years later (in 2011). Further, we aim to validate latent profiles of maternal mental health problems previously identified using the same data material [35, 36]. The trajectories identified were 1) “No/Low” group with zero or low symptom levels; 2) “Moderate” group with moderate symptom scores; 3) “High” group with overall high symptom levels; and 4) “Low-Rising” group with initially low symptom levels that increased to a level similar to the High group. The study expands upon previous studies by using a longer time span and specifically focusing on employed women with children throughout the child-rearing period. Expanding on former studies of mental health trajectories, we also adjust for age, negative trait-based personality (e.g., negative emotionality) and job demands, which have been found to be linked to both mental health, burnout and work-family conflict [14, 20]. We expected the three adverse mental health trajectories (i.e., High, Moderate and Low-rising) to predict subsequent higher levels of burnout and work-family conflict than the Low group, with the highest levels for the High trajectory group.

## Method

### Design

The current study used data from the Tracking Opportunities and Problems (TOPP) - study, a community-based longitudinal study [37]. The study has been approved by The Data Inspectorate and the Regional Committee for Medical Research Ethics. Study participation was voluntary and families were given written information before and after each data collection about current and future research, the confidentiality of the participants, the possibility to skip questions, and the right to withdraw from the study at any point. All families from 19 geographic health care areas in Eastern Norway were invited to complete a survey questionnaire when attending the 18-month vaccination visit for their child. Routinely, 90–95 % of all Norwegian families with children attend a public health program during the first 4 years of the child’s life. Of 1081 invited families at the child health clinic, 913 women participated at baseline. The women received survey questionnaires at eight data collections between 1993 and 2011, when their children were 1.5 (*n* = 913 (1993), 2.5 years (*n* = 777 (1994)), 4.5 years (*n* = 727 (1996)), 8.5 years (*n* = 505 (2000)), 12.5 years (*n* = 587 (2004)), 14.5 years (*n* = 472 (2006)), 16.5 years (*n* = 421 (2008)) and 19 years (*n* = 519 (2011)). The questionnaires were administered at the child health clinics for the first three waves and by post from the fourth wave.

### Sample

The sample for the current study included employed women (*n* = 447) with data on trajectories of mental health between 1993 and 2006, and outcomes (e.g., burnout and work-family conflict) in 2011. The outcomes were only available in 2011. In 2011, 13.5 % (*n* = 70) of women were either students, home-staying parents, on disability pension or seeking jobs and were excluded due to the nature of the burnout and work-family items (i.e., asking about conditions at work). The degree of missing data was low, and only two women were excluded due to missing data on the outcome variables. The age of the participating women ranged from 19 to 46 years (mean = 30; SD: 4.7) at baseline (1993) to 38–61 years (mean = 49.44; SD = 4.63) in 2011. The average working hours varied from 6 to 70 h per week (mean = 35.98; SD = 8.49), and 30 % of the women had management responsibilities in 2011.

### Attrition

Background data from the child health clinics showed that non-responding women did not differ significantly from responding women with regard to maternal age, education, employment, or marital status at baseline [37]. Attrition analyses examining women from baseline to the fifth wave [38] and the seventh wave [39], revealed no differences in drop-out versus remaining families in several traits (e.g., temperament and mental health problems, and support from partner and friends). However, women who dropped out from the study had lower educational levels than non-dropout women. The associations between variables at baseline compared to the last waves of the study did not differ among drop-out versus remaining families, suggesting that estimated associations between variables are generalizable [39].

### Measures

#### Predictor: trajectories of mental health problems

Mental health problems were measured at every wave (1993–2011) with symptoms of anxiety and depression (hereafter referred to as “mental health problems”) from the Hopkins Symptoms Check List (SCL) [40, 41], with four response categories (“Not at all”, “A little”, “Quite a bit”, and “Very much”). The SCL has shown satisfactory psychometric qualities in earlier studies [42–44].

The original version consists of 25 items. In the current survey, one item was removed at the first two waves (“Thoughts of killing yourself”) and another item (“Loss of sexual interest or pleasure”) was removed at all waves, because it was perceived as offensive in the pilot-study [37]. A 10-item version of SCL was used at the fifth time point for a shorter questionnaire to raise response rates. Findings comparing various short versions of the SCL found all versions to show almost equally high internal consistency, sensitivity and specificity [44–46]. Because the 10- and 24-item versions correlated highly with each other and because we did not want to lose variance by using 10-item versions at each time point, all available information was used for each time point to identify latent trajectories [35, 36]. The internal reliability in the current study was high at every time points, ranging from *α* = .87 to *α* = .91.

The current study is based on formerly identified latent trajectories (or profiles) based on SCL from 1993 to 2006 with latent profile analysis (LPA) by Skipstein et al. [35, 36]. LPA is a person-centered approach which groups the sample into a set of latent subpopulations with similar developmental trajectories [47]. Posterior class membership probabilities were estimated after model estimation for the assignment of individuals to pseudo-classes according to the maximum probability rule [35, 48]. Despite not taking into consideration the uncertainty of the class memberships when using pseudo-classes, the average posterior class probabilities for trajectory membership for the group solution ranged from .80 to .91. Thus, the ambiguities regarding the class memberships were not large and the effect of not including the uncertainty of latent class membership in the analyses is thus not likely to produce substantial bias to the current results [35, 36]. For the current study, four identified trajectories from 1993 to 2006 were used. These are defined as: 1) “No/Low” group with zero or low symptom levels (*n* = 252; 56.4 %); 2) “Moderate” group with moderate symptom scores (*n* = 143; 32 %); 3) “High” group with overall high symptom levels (*n* = 34; 7.6 %); and 4) “Low-Rising” group with initially low symptom levels in 1993 that starts to increase from 2000 to reach a level similar to the High group by 2011 (*n* = 18; 4 %) (For more details: [35, 36]).

#### Outcomes: burnout and work-family conflict

Burnout was measured with the Oldenburg Burnout Inventory (OLBI) in 2011 [21, 22], consisting of two subscales measuring exhaustion and disengagement from work to be scored from “1” (strongly disagree) to “4” (strongly agree). Exhaustion consisted of seven items, such as “After my work, I regularly feel worn out and weary” and disengagement consisted of eight items, such as “I frequently talk about my work in a negative way”. The Cronbach Alpha was .81–.85, for exhaustion and disengagement from work, respectively. A mean score for each subscale was calculated.

Work-family conflict was measured by two items in 2011: “Problems adjusting work with child care” and “Miss spending time with the children/adolescents” from a chronic stress scale with responses ranging from “0” (no stress) to “3” (very high stress) [37, 38]. The instrument was not expected to exhibit high reliability given its formative nature as well as the few items examined. A mean score was calculated.

### Covariates

Women’s self-reported age, job demands, negative emotionality and mental health problems measured in 2011 were controlled for. Job demands were measured by a mean score of five items (e.g., “How often do you perform work with constant time pressure because of high work load”) from the Job Content Questionnaire [49]. The Cronbach alpha was satisfactory (.77). Negative emotionality was measured by a mean score of 12 items from the Emotionality, Activity and Sociability scale (EAS) for adults [50, 51]. Example items are “I get emotionally upset easily” and “I often feel insecure”. Cronbach alpha was 0.80. The factor structure and psychometric properties of this scale have previously been assessed in this sample and shown to be satisfactory [51]. We used the mean score of the SCL scale to measure mental health in 2011 as control variable. The cronbach alpha was .91.

### Statistical analyses

A one-way between-subjects multivariate analyses of variance with IBM SPSS Statistics 23 was used to assess the association between the four trajectory groups measured across six time points between 1993 and 2006, and subsequent burnout (i.e., exhaustion and disengagement from work) in 2011. Similarly, a one-way between-subjects univariate analysis of variance was used to assess the association between the four trajectory groups and subsequent work-family conflict in 2011. Analyses were conducted and depicted in a table by stepwise adjustments: 1) No adjustments; 2) Age; 3) Job demands; 4) Negative emotionality; and 5) Concurrent mental health problems.

## Results

Table 1 shows the correlations between mental health problem at each wave between 1993 and 2006, and burnout and work-family conflict in 2011. All cross-sectional and longitudinal associations were significantly correlated.

**Table 1:** Pearson’s correlations between mental health scores in 1993–2006 and work-related outcomes (exhaustion, disengagement and work-family conflict) in 2011 in employed Norwegian women with children

	Work-related outcomes in 2011		
	Exhaustion	Disengagement	Work-family conflict
Mental health scores (1993–2006)			
T1 (1993)	0.27^c^	0.21^c^	0.20^c^
T2 (1994)	0.23^c^	0.17^b^	0.17^b^
T3 (1996)	0.31^c^	0.24^c^	0.16^b^
T4 (2000)	0.32^c^	0.26^c^	0.29^c^
T5 (2004)	0.31^c^	0.24^c^	0.13^a^
T6 (2006)	0.36^c^	0.28^c^	0.13^a^
Outcomes 2011			
Exhaustion			
Disengagement	0.61^c^		
Work-family conflict	0.23^c^	0.14^b^	

^a^Correlation is significant at the 0.05 level (2-tailed)

^b^Correlation is significant at the 0.01 level (2-tailed)

^c^Correlation is significant at the 0.001 level (2-tailed)

One-way between-subjects multivariate analyses of variance were carried out to assess the impact of different trajectories of mental health problems (i.e., four different trajectories) on burnout (i.e., exhaustion and disengagement from work). There was a statistically significant difference between the four trajectories on burnout: F (6,878) = 10.93, *p* = .000, Wilks’ Lambda = 0.87, partial *η* ^2^ = .07. There were significant group differences for both exhaustion: F (3,440) = 21.64, *p* = .000, and disengagement from work: F (3,440) = 11.67, *p* = .000. The trajectories explained 13 and 7 % of the variance in exhaustion and disengagement measured by partial *η* ^2^.

Employing Bonferroni corrected post-hoc tests, the High, Moderate and Low-Rising groups had higher scores on exhaustion and disengagement, versus the Low group (*p* = .000). There were no significant group differences between the Moderate, High and Low-Rising groups on either exhaustion or disengagement from work (*p* > .05). The Low-Rising group scored higher on exhaustion versus the Low group (*p* = .000). The mean scores as well as confidence intervals are reported in Tables 2 and 3. The findings remained the same when adjusting for age, emotionality and job demands, except for the association between the Low-Rising group and burnout which disappeared.

**Table 2:** Mean scores and Confidence Intervals (CI) in exhaustion (2011) for different trajectories of mental health (1993–2006) among employed Norwegian women with children

Mental health trajectory groups^a^	Model 1^b^	Model 2^c^	Model 3^d^	Model 4^e^	Model 5^f^
	Mean CI 95 %	Mean CI 95 %	Mean CI 95 %	Mean CI 95 %	Mean CI 95 %
Low	1.88 (1.82–1.93)	1.88 (1.82–1.93)	1.92 (1.87–1.98)	1.87 (1.83–1.93)	1.95 (1.90–2.01)
Moderate	2.16^g^ (2.09–2.24)	2.16^g^ (2.09–2.24)	2.13^g^ (2.06–2.20)	2.16^g^ (2.09–2.23)	2.11^g^ (2.04–2.18)
High	2.34^g^ (2.20–2.49)	2.34^g^ (2.20–2.49)	2.24^g^ (2.09–2.39)	2.34^g^ (2.18–2.47)	2.08 (1.93–2.23)
Low–Rising	2.21^g^ (2.01–2.42)	2.21^g^ (2.01–2.42)	2.10 (1.90–2.29)	2.21^g^ (2.01–2.41)	2.11 (1.92–2.30)

^a^Four different trajectories of mental health levels from 1993 and 2006: 1) “Low “: Consistent low levels, 2) “Moderate”: Consistent moderate levels, 3) “High”: Consistent high levels, and 4) “Low-rising”: Low levels that rise to high levels over the 13-year period

^b^Model 1: Unadjusted

^c^Model 2: Adjusted for age

^d^Model 3: Adjusted for negative emotionality

^e^Model 4: Adjusted for job demands

^f^Model 5: Adjusted for mental health problems at T8

^g^Significantly different from the Low Mental Health-trajectory group

**Table 3:** Mean scores and Confidence Intervals (CI) in disengagement from work (2011) for different trajectories of mental health (1993–2006) among employed Norwegian women with children

Mental health trajectory groups^a^	Model 1^b^	Model 2^c^	Model 3^d^	Model 4^e^	Model 5^f^
	Mean CI 95 %	Mean CI 95 %	Mean CI 95 %	Mean CI 95 %	Mean CI 95 %
Low	1.95 (1.89–2.01)	1.95 (1.89–2.01)	1.99 (1.93–2.05)	1.95 (1.89–2.00)	2.02 (1.96–2.08)
Moderate	2.17^g^ (2.08–2.25)	2.16^g^ (2.08–2.24)	2.13^g^ (2.05–2.21)	2.16^g^ (2.08–2.24)	2.11 (2.03–2.19)
High	2.36^g^ (2.20–2.53)	2.36^g^ (2.19–2.52)	2.27^g^ (2.10–2.43)	2.38^g^ (2.22–2.54)	2.12 (1.94–2.29)
Low-Rising	2.26^g^ (2.03–2.48)	2.26^g^ (2.03–2.49)	2.15 (1.92–2.37)	2.26^g^ (2.04–2.49)	2.16 (1.94–2.38)

^a^Four different trajectories of mental health levels from 1993 and 2006: 1) “Low “: Consistent low levels, 2) “Moderate”: Consistent moderate levels, 3) “High”: Consistent high levels, and 4) “Low-rising”: Low levels that rise to high levels over the 13-year period

^b^Model 1: Unadjusted

^c^Model 2: Adjusted for age

^d^Model 3: Adjusted for negative emotionality

^e^Model 4: Adjusted for job demands

^f^Model 5: Adjusted for mental health problems at T8

^g^Significantly different from the Low Mental Health-trajectory group

When adjusting for concurrent mental health problems (2011), there were no longer significant differences between the four trajectories on the burnout variables in total: F (6,876) = 2.04, *p* = .058, Wilks’ Lambda = 0.97, partial *η* ^2^ = .01. There were significant differences for the trajectory groups for exhaustion: F (3,439) = 4.03, *p* = .008, partial *η* ^2^ = .03, but not for disengagement from work: F (3,439) = 1.24, *p* = .293, partial *η* ^2^ = .01. The Moderate group scored significantly higher than the Low group on exhaustion.

There was a statistically significant difference between the four trajectories on work-family conflict: F (3,443) = 7.02, *p* = .000. The trajectories accounted for 5 % of the variance in work-family conflict measured by partial *η* ^2^. Employing Bonferroni-corrected post-hoc tests to examine differences between the trajectory groups revealed that women in the High and the Moderate group scored significantly higher on work-family conflict versus the Low distress group. There were no significant differences between the Low-Rising and the other groups. The mean scores are reported in Table 4. When adjusting for age, emotionality, and job demands the findings remained significant. However, when adjusting for concurrent maternal depression, there were no longer any significant effects of any of the trajectory groups on work-family conflict.

**Table 4:** Mean scores and Confidence Intervals (CI) in work-family conflict (2011) for different trajectories of mental health (1993–2006) among employed Norwegian women with children

Mental health trajectory groups^a^	Model 1^b^	Model 2^c^	Model 3^d^	Model 4^e^	Model 5^f^
	Mean CI 95 %	Mean CI 95 %	Mean CI 95 %	Mean CI 95 %	Mean CI 95 %
Work-family conflict					
Low	0.43 (0.37–0.48)	0.43 (0.38–0.49)	0.45 (0.39–0.51)	0.43 (0.37–0.48)	0.46 (0.40–0.52)
Moderate	0.57^g^ (0.49–0.64)	0.56^g^ (0.49–0.63)	0.56^g^ (0.48–0.63)	0.57^g^ (0.50–0.64)	0.55 (0.47–0.63)
High	0.76^g^ (0.61–0.92)	0.75^g^ (0.60–0.90)	0.72^g^ (0.56–0.88)	0.74^g^ (0.59–0.89)	0.67 (0.50–0.84)
Low-Rising	0.47 (0.26–0.68)	0.48 (0.27–0.69)	0.42 (0.21–0.63)	0.47 (0.26–0.67)	0.43 (0.22–0.64)

^a^Four different trajectories of mental health levels from 1993 and 2006: 1) “Low “: Consistent low levels, 2) “Moderate”: Consistent moderate levels, 3) “High”: Consistent high levels, and 4) “Low-rising”: Low levels that rise to high levels over the 13-year period

^b^Model 1: Unadjusted

^c^Model 2: Adjusted for age

^d^Model 3: Adjusted for negative emotionality

^e^Model 4: Adjusted for job demands

^f^Model 5: Adjusted for mental health problems at T8

^g^Significantly different from the Low Mental Health-trajectory group

## Discussion

This is the first study following employed mothers over 18 years of the child rearing period to examine mental health trajectories, burnout and work-family conflict. Based on a person-centered approach, we examined if different trajectories of mental health across 13 years predicted subsequent work- and family-related outcomes﻿. We found that adverse trajectories of mental health was a valid predictor of subsequent burnout and work-family conflict, even after controlling for age, negative emotionality and job demands.

Women experiencing an adverse trajectory of mental health problems (Moderate, High or Low-rising levels) between 1993 and 2006 had significantly higher levels of work- and family related consequences in 2011. Having adverse symptom trajectories over this period accounted for 7–13 % of the variance in burnout (e.g., exhaustion and disengagement from work) and 5 % of work-family conflict. Although explaining a small portion of the variance, this indicates that adverse patterns of mental health problems already present early in the child-rearing process gives a heightened risk for subsequent adverse work-related outcomes - especially for those with consistently high or moderate levels of mental health problems. Because the time period spans over 18 years, a larger proportion of explained variance was not expected. Moreover, the associations were robust when accounting for individual characteristics, such as age, negative emotionality, and work characteristics, which have been found to relate to mental health, burnout and work-family conflict [14, 20].

The association between mental health and work- and family outcomes is especially interesting considering that the current sample consists of “normal-functioning” families. The implications are that one should early pay attention to those categorized as scoring both high or moderate levels of depressive symptoms during the child-rearing period. Not surprisingly when the time span stretches over a period of 18 years with a lag of five years, the small amount of variance explained suggests that there are other factors affecting burnout and work-family conflict in addition to mental health problems. In a former study using the same data, Skipstein et al. [35, 36] reported that women reporting consistently high mental health problems (i.e., High trajectory group) were younger, had lower education, and fewer were living with a partner compared to the women with less mental health problems. This might contribute to an understanding of who are at increased risk of long-term work- and family-related adverse outcomes. Moreover, former studies indicate that consistently high mental health problems over time are related to maladaptive coping strategies [32]. Future studies could examine coping as a potential protective factor or mechanism behind the link between consistent mental health problems and work- and family-related adversities.

Our findings support formerly suggested links between mental health and burnout [30]. For instance, a 10-year longitudinal study of university students reported high depressive symptom trajectories to be associated with subsequent burnout compared to moderate and low symptom trajectories [32]. The term burnout has been criticized to some extent to be conceptually too similar to mental health problems [30]. Of note, our findings indicate, that although the effects of consistently high and low-rising patterns of mental health symptoms was eliminated when adjusting for concurrent mental health problems, the effect of consistently moderate symptom levels 5 years later persisted. However, this was only found for exhaustion, and not for disengagement from work. Former findings have indeed reported differences between exhaustion and disengagement from work. For instance, Peterson et al. [24] reported that the exhaustion dimension of the OLBI burnout instrument, which was also used in the current study, was a better predictor of long-term sickness absence compared to disengagement, depression and anxiety. Our findings further highlight the link between exhaustion and former history of mental health problems, indicating that exhaustion is the most detrimental symptom of burnout.

Our findings indicate that consistent moderate and high adverse patterns of mental health problems across the child-rearing period contribute to conflict between work and family. Noteworthy, even when looking at crude correlations, mental health scores at the first time points (1993 and 1994) were significantly associated with all examined outcomes from two to 18 subsequent years later. Despite replicating former published findings on the relations between mental health and work-family conflict [15, 16, 31], it is somewhat surprising to also find these associations in a sample of women living in a context of high gender egalitarianism and a generous welfare system promoting flexible work-family solutions, long parental leaves and other social benefits. Even though not explicitly examined, the current study does not offer support for the notion that higher levels of support from the state, workplace and family buffer the potential negative effect of work-life imbalance for those who have moderate symptoms, as suggested in former publications [34]. The frequency of employed women with children is however higher in Norway compared to other countries, with high cultural expectations of combining work and family. With higher labour force participation – despite more flexible work-family solutions, there might be a higher portion of women with mental health problems in the labour force.

Those experiencing consistent moderate mental health problems over the entire child-rearing period had increased risk for exhaustion even over and above concurrent mental health problems. This highlights the importance of preventing mental health problems – even on moderate levels – as it not only affects the work arena, but also the family arena. Since both mother’s mental health problems and work-family conflict are related to parenting skills [28], targeting even those with moderate levels of mental health problems might prevent adverse intergenerational effects. Future studies should examine the interplay between mental health and work-family conflict and other important partner- and child-related variables (e.g., parenting skills and relationship satisfaction) in order to gain knowledge that can be used in policy making and prevention efforts before cross-over effects to other family members can occur.

### Strengths and limitations

Although the current study has several strengths such as its longitudinal approach following employed women with children over 18 years, there are also some methodological and conceptual limitations. First, the use of self-report measures increases the risk of bias. However, using multiple waves reduces common method bias as well as daily fluctuations in mood. When we adjust for negative emotionality, as well as concurrent mental health problems, we also adjust for the potential depression-distortion effect (i.e., depression might make the perception of other factors, such as burnout, more negative) to a certain degree. Using a population-based sample to examine mental health also reduces the selection bias associated with clinical samples. Although clinical diagnostic interviews would increase the clinical validity of the study, self-reported mental health problems are considered to be valid pointer when gaining knowledge from high prevalent symptoms within a population [52, 53]. Related to this, although burnout is measured by a psychometrically valid instrument [21, 22], the work-family conflict measure consisted of two self-made questions. Although we believe the measure has satisfactory face validity and thus captures a meaningful part of the aspect of work-family conflict, using a more valid measure consisting of more items would perhaps have captured stronger associations.

Second, the patterns of drop-outs in the current sample propose some caution in generalizing the findings. The response rate was 56 % at the final wave in 2011. Attrition rates as high as 40–60 % are not uncommon in longitudinal studies and only systematic non-random drop-outs represent a problem [54]. The only systematic difference between drop-outs and remaining participants was low level of education [38, 39]. Still, one possible bias when analyzing attrition rates based on former data waves is the notion that drop-outs are not static, i.e., drop-outs from the first waves, who are believed not to be different from respondents, may have changed after dropping out of the study. For instance, participants’ stress levels at the second wave may have increased by the third wave. This is often ignored when reporting drop-out analyses. However, associations between variables at baseline did not differ among drop-out versus remaining families later in the TOPP-study, indicating that estimated associations between variables are generalizable [39]. This suggests that on one side significant findings in our study may be stronger in a high risk sample (i.e., those with low levels education), since there might be more variation in the responses (for instance to mental health problems), but on the other side; null-findings must be interpreted with care.

Related to this, caution must also be taken in generalizing the findings to non-parents as there are some controversies as to which parents have less or more mental health problems. On the one hand, research suggests that parents have more mental health problems due to increased daily stress associated with child-rearing [55–57]. On the other hand, non-parents, despite less potential to experience stress associated with having children, might be at risk of other stressors such as being lonely, and thus report higher levels of mental health problems which have been found in some studies [56]. Other studies have reported no difference between parents and non-parents with regards to mental health problems/disorders [4, 56, 58, 59]. Despite the needed caution in generalizing the findings to non-parents, examining mental health in employed parents is important due to their caregiving role, the potential intergenerational transmission and the lack of studies with a family design within work-related outcomes.

Finally, we did not have the opportunity to adjust for burnout or work-family conflict at earlier time points. The women with adverse mental health trajectories might have experienced consistent levels of work-family conflict or burnout throughout the period, which could be the reason for the adverse trajectory of mental health. In fact, findings have suggested reciprocal effects between work-family conflict and mental health problems in employed mothers with children followed in early childhood [31]. Former findings using the same data suggest that high levels of child-related stress predict higher levels of mental health problems [35–37, 51]. Future studies should examine the interplay between parent and child factors to delineate the indirect paths across time for families across the child-rearing period to see at what times families are most vulnerable to experience mental health, burnout and/or work-family conflict.

## Conclusions

Our study revealed that reduced mental health of employed mothers during the child-rearing period - even at moderate levels - are relevant predictors of future burnout and work-family conflict, which are important indicators of how well an individual is functioning at work and at home. Therefore, our message for policy makers and organizations is to keep track of the mental health of women during this period of life in order to avoid the development of problems in a wide range of life domains as well as intergenerational transmission.

## References

[1] Burke L (2003). The impact of maternal depression on familial relationships. Int Rev Psychiatry.

[2] Goodman SH (2007). Depression in mothers. Annu Rev Clin Psychol.

[3] Kessler RC, Wang PS, Gotlib IHHC (2009). The epidemiology of depression. Handbook of depression.

[4] Davila J, Stroud CB, Starr LR, Gotlib IH, Hammen CL (2009). Depression in couples and families. Handbook of depression.

[5] Nelson SK, Kushlev K, Lyubomirsky S (2014). The pains and pleasures of parenting: when, why, and how is parenthood associated with more or less well-being?. Psychol Bull.

[6] Chang JJ, Halpern CT, Kaufman JS (2007). Maternal depressive symptoms, father’s involvement, and the trajectories of child problem behaviors in a US national sample. Arch Pediatr Adolesc Med.

[7] Muthen B, Muthen LK (2000). Integrating person-centered and variable-centered analyses: growth mixture modeling with latent trajectory classes. Alcohol Clin Exp Res.

[8] Laursen B, Hoff E (2006). Person-centered and variable-centered approaches to longitudinal data. Merrill Palmer Quart.

[9] Stoolmiller M, Kim HK, Capaldi DM (2005). The course of depressive symptoms in men from early adolescence to young adulthood: identifying latent trajectories and early predictors. J Abnorm Psychol.

[10] Borritz M, Rugulies R, Christensen KB, Villadsen E, Kristensen TS (2006). Burnout as a predictor of self-reported sickness absence among human service workers: prospective findings from three year follow up of the PUMA study. Occup Environ Med.

[11] Honkonen T, Ahola K, Pertovaara M, Isometsa E, Kalimo R, Nykyri E, Aromaa A, Lonnqvist J (2006). The association between burnout and physical illness in the general population--results from the Finnish Health 2000 Study. J Psychosom Res.

[12] Taris TW (2006). Is there a relationship between burnout and objective performance? A critical review of 16 studies. Work Stress.

[13] Deligkaris P, Panagopoulou E, Montgomery AJ, Masoura E (2014). Job burnout and cognitive functioning: a systematic review. Work Stress.

[14] Amstad FT, Meier LL, Fasel U, Elfering A, Semmer NK (2011). A meta-analysis of work-family conflict and various outcomes with a special emphasis on cross-domain versus matching-domain relations. J Occup Health Psychol.

[15] Eby LT, Casper WJ, Lockwood A, Bordeaux C, Brinley A (2005). Work and family research in IO/OB: content analysis and review of the literature (1980–2002). J Vocat Behav.

[16] Allen TD, Herst DE, Bruck CS, Sutton M (2000). Consequences associated with work-to-family conflict: a review and agenda for future research. J Occup Health Psychol.

[17] Ford MT, Heinen BA, Langkamer KL (2007). Work and family satisfaction and conflict: a meta-analysis of cross-domain relations. J Appl Psychol.

[18] Kossek EE, Ozeki C (1998). Work-family conflict, policies, and the job-life satisfaction relationship: a review and directions for organizational behavior human resources research. J Appl Psychol.

[19] Kossek EE, Noe RA, DeMarr BJ (1999). Work-family role synthesis: individual and organizational determinants. Int J Confl Manage.

[20] Maslach C, Schaufeli WB, Leiter MP (2001). Job burnout. Annu Rev Psychol.

[21] Demerouti E, Mostert K, Bakker AB (2010). Burnout and work engagement: a thorough investigation of the independency of both constructs. J Occup Health Psychol.

[22] Demerouti E, Bakker AB, Vardakou I, Kantas A (2003). The convergent validity of two burnout instruments: a multitrait-multimethod analysis. Eur J Psychol Assess.

[23] Freudenberger HJ (1974). Staff burn-Out. J Soc Issues.

[24] Peterson U, Bergstrom G, Demerouti E, Gustavsson P, Asberg M, Nygren A (2011). Burnout levels and self-rated health prospectively predict future long-term sickness absence: a study among female health professionals. J Occup Environ Med.

[25] Greenhaus JH, Allen TD, Quick JC, Tetric LE (2011). Work-family balance: a review and extension of the literature. Handbook of occupational health psychology.

[26] Greenhaus JH, Beutell NJ (1985). Sources of conflict between work and family roles. Acad Manage Rev.

[27] Greenhaus JH, Parasuraman S (1986). Vocational and organizational behavior, 1985: a review. J Vocat Behav.

[28] Cooklin AR, Westrupp E, Strazdins L, Giallo R, Martin A, Nicholson JM (2015). Mothers’ work-family conflict and enrichment: associations with parenting quality and couple relationship. Child Care Health Dev.

[29] Frone MR, Yardley JK, Markel KS (1997). Developing and testing an integrative model of the work-family interface. J Vocat Behav.

[30] Bianchi R, Schonfeld IS, Laurent E (2015). Burnout-depression overlap: a review. Clin Psychol Rev.

[31] Westrupp EM, Strazdins L, Martin A, Cooklin A, Zubrick SR, Nicholson JM. Maternal work–family conflict and psychological distress: reciprocal relationships over 8 years. J Marriage Fam. 2015.

[32] Salmela-Aro K, Aunola K, Nurmi JE (2008). Trajectories of depressive symptoms during emerging adulthood: antecedents and consequences. Eur J Dev Psychol.

[33] Eikemo TA, Bambra C (2008). The welfare state: a glossary for public health. J Epidemiol Community Health.

[34] Abendroth AK, den Dulk L (2011). Support for the work-life balance in Europe: the impact of state, workplace and family support on work-life balance satisfaction. Work Employ Soc.

[35] Skipstein A, Janson H, Stoolmiller M, Mathiesen KS (2010). Trajectories of maternal symptoms of anxiety and depression. A 13-year longitudinal study of a population-based sample. BMC Public Health.

[36] Skipstein A, Janson H, Kjeldsen A, Nilsen W, Mathiesen KS (2012). Trajectories of maternal symptoms of depression and anxiety over 13 years: the influence of stress, social support, and maternal temperament. BMC Public Health.

[37] Mathiesen KS, Tambs K, Dalgard OS (1999). The influence of social class, strain and social support on symptoms of anxiety and depression in mothers of toddlers. Soc Psych Psych Epid.

[38] Karevold E, Roysamb E, Ystrom E, Mathiesen KS (2009). Predictors and pathways from infancy to symptoms of anxiety and depression in early adolescence. Dev Psychol.

[39] Gustavson K, von Soest T, Karevold E, Roysamb E (2012). Attrition and generalizability in longitudinal studies: findings from a 15-year population-based study and a Monte Carlo simulation study. BMC Public Health.

[40] Hesbacher PT, Rickels K, Morris RJ, Newman H, Rosenfeld H (1980). Psychiatric illness in family practice. J Clin Psychiatry.

[41] Winokur A, Winokur DF, Rickels K, Cox DS (1984). Symptoms of emotional distress in a family planning service: stability over a four-week period. Br J Psychiatry.

[42] Deane FP, Leathern J, Spicer J (1992). Clinical norms, reliability and validity for the hopkins symptom checklist-21. Aust J Psychol.

[43] Nettelbladt P, Hansson L, Stefansson CG, Borgquist L, Nordstrom G (1993). Test characteristics of the Hopkins Symptom Check List-25 (HSCL-25) in Sweden, using the Present State Examination (PSE-9) as a caseness criterion. Soc Psych Psych Epid.

[44] Tambs K, Moum T (1993). How well can a few questionnaire items indicate anxiety and depression?. Acta Psychiatr Scand.

[45] Strand BH, Dalgard OS, Tambs K, Rognerud M (2003). Measuring the mental health status of the Norwegian population: a comparison of the instruments SCL-25, SCL-10, SCL-5 and MHI-5 (SF-36). Nord J Psychiatry.

[46] Müller J, Postert C, Beyer T, Furniss T, Achtergarde S (2010). Comparison of eleven short versions of the symptom checklist 90-revised (SCL-90-R) for use in the assessment of general psychopathology. J Psychopathol Behav Assess.

[47] Lincoln KD, Chatters LM, Taylor RJ, Jackson JS (2007). Profiles of depressive symptoms among African Americans and Caribbean Blacks. Soc Sci Med.

[48] Hipp JR, Bauer DJ (2006). Local solutions in the estimation of growth mixture models. Psychol Methods.

[49] Karasek R, Brisson C, Kawakami N, Houtman I, Bongers P, Amick B (1998). The Job Content Questionnaire (JCQ): an instrument for internationally comparative assessments of psychosocial job characteristics. J Occup Health Psychol.

[50] Buss AH, Plomin R (1984). Temperament: early developing personality traits.

[51] Naerde A, Roysamb E, Tambs K (2004). Temperament in adults--reliability, stability, and factor structure of the EAS Temperament Survey. J Pers Assess.

[52] Mayberry LJ, Horowitz JA, Declercq E (2007). Depression symptom prevalence and demographic risk factors among U.S. women during the first 2 years postpartum. J Obstet Gynecol Neonatal Nurs.

[53] Sandanger I, Moum T, Ingebrigtsen G, Dalgard OS, Sorensen T, Bruusgaard D (1998). Concordance between symptom screening and diagnostic procedure: the Hopkins Symptom Checklist-25 and the Composite International Diagnostic Interview I. Soc Psychiatry Psychiatr Epidemiol.

[54] van der Kamp LJT, Bijleveld CCJH. Methodological issues in longitudinal research. In: Longitudinal data analysis: designs, models and methods. edn. Edited by C.C.J.H. Bijleveld LJTvdK, A. Mooijaart, W. A. van der Kloot, R. van der Leeden, & E. Van Der Burg London, England: Sage; 1998: 1–44.

[55] Evenson RJ, Simon RW (2005). Clarifying the relationship between parenthood and depression. J Health Soc Behav.

[56] Rimehaug T, Wallander J (2010). Anxiety and depressive symptoms related to parenthood in a large Norwegian community sample: the HUNT2 study. Soc Psychiatry Psychiatr Epidemiol.

[57] Munk-Olsen T, Laursen TM, Pedersen CB, Mors O, Mortensen PB (2006). New parents and mental disorders: a population-based register study. JAMA.

[58] van Bussel JC, Spitz B, Demyttenaere K (2006). Women’s mental health before, during, and after pregnancy: a population-based controlled cohort study. Birth (Berkeley, Calif).

[59] Olson AL, Dietrich AJ, Prazar G, Hurley J (2006). Brief maternal depression screening at well-child visits. Pediatrics.

